# Mathematical Models for Cholera Dynamics—A Review

**DOI:** 10.3390/microorganisms10122358

**Published:** 2022-11-29

**Authors:** Jin Wang

**Affiliations:** Department of Mathematics, University of Tennessee at Chattanooga, Chattanooga, TN 37403, USA; jin-wang02@utc.edu

**Keywords:** mathematical modeling, cholera, disease transmission, intervention

## Abstract

Cholera remains a significant public health burden in many countries and regions of the world, highlighting the need for a deeper understanding of the mechanisms associated with its transmission, spread, and control. Mathematical modeling offers a valuable research tool to investigate cholera dynamics and explore effective intervention strategies. In this article, we provide a review of the current state in the modeling studies of cholera. Starting from an introduction of basic cholera transmission models and their applications, we survey model extensions in several directions that include spatial and temporal heterogeneities, effects of disease control, impacts of human behavior, and multi-scale infection dynamics. We discuss some challenges and opportunities for future modeling efforts on cholera dynamics, and emphasize the importance of collaborations between different modeling groups and different disciplines in advancing this research area.

## 1. Introduction

Cholera is an infectious disease caused by the bacterium *Vibrio cholerae* (or, *V. cholerae*) [1]. The main sources of the pathogen are contaminated water and food. The infection can spread rapidly in populations without safe drinking water and adequate sanitation and hygiene, and those with limited medical resources [2]. The major symptom of cholera is profuse watery diarrhea, which could result in rapid dehydration. Other symptoms may include vomiting, extreme thirst, abdominal pain, kidney failure, and drop in blood pressure. In the most severe cases, cholera can lead to death within days if not treated [3].

Although cholera is an old disease, with seven pandemics already recorded in human history, the global burden of cholera remains high at present. This is largely due to lack of access to basic drinking water and sanitation in many places of the world. It was estimated that more than 2 billion people worldwide drink water from sources that may be faecally contaminated, and 2.4 billion people do not have basic sanitation facilities, exposing them to cholera and other waterborne infections [4]. From a report published in 2015 [5], it was found that approximately 1.3 billion people were at risk for cholera in endemic countries and regions, and about 1.3–4.0 million people were infected with cholera annually, including an estimated 95,000 deaths. More recently, Yemen experienced the worst cholera outbreak in modern history that started in late 2016 and peaked in 2017, with more than 2.5 million cumulative cases reported as of November 2021 [6]. In October 2017, the World Health Organization (WHO) launched a global strategy for ending cholera, with an aim of reducing cholera deaths by 90% and eliminating cholera in 20 of the 47 countries currently affected by the disease by 2030 [7]. Ultimately, the improvement of water, sanitation and hygiene (WASH) infrastructure is the fundamental, long-term solution for cholera control [2].

Mathematical modeling for infectious diseases dates back to Bernoulli in the 18th century [8], and has since become indispensable in epidemiological research [9]. Models offer a powerful theoretical tool to understand infection and transmission mechanisms, to predict future progression of epidemics, to compare different intervention strategies, and to provide useful guidelines for outbreak management [10].

**Figure 1:**
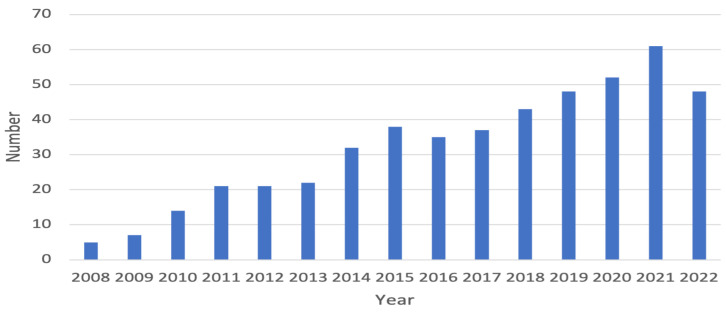
Number of published cholera-modeling studies by year. Search conducted in October 2022 based on Google Scholar.

Numerous mathematical models have been developed to study cholera dynamics. In order to have an understanding of the volume of research activity in this area, we conducted a simple search in October 2022 using Google Scholar for the number of cholera-modeling studies published in the last 15 years (2008–2022). The search was based on the title of any article containing the keyword “cholera” and at least one of the following keywords: “mathematical”, “model”, “models”, “modeling”, “modelling”, and “dynamics”. The records returned by Google Scholar were then individually screened, duplicates were identified and removed, and those items not related to mathematical modeling were also removed. The final search results were summarized in Figure 1. Our search was not meant to be exhaustive, as it excluded those cholera-modeling studies that do not have one of those aforementioned keywords in their titles and those that are not indexed by Google Scholar. Nevertheless, even with this incomplete search, we have found almost 500 pieces of modeling work for cholera published over the last 15 years, as shown in Figure 1. We see a clear pattern that the number of articles for cholera modeling has been fast growing, with an increase of more than 10 times from 2008 to 2021.

Two excellent reviews of mathematical modeling for cholera were published in 2014 by Chao et al. [11] and Fung [12], both targeting the public health community, with a special emphasis on modeling work devoted to the 2010 Haiti cholera outbreak. Eight years have passed since the publication of these two reviews, and a large number of new modeling studies for cholera have since appeared (see Figure 1). The main goal of the current article is to review the state of the art in mathematical modeling studies for cholera that include both the earlier development (before 2014) and the new progress (after 2014). A special effort is made to survey a wide range of modeling techniques that have been employed for cholera dynamics, including such topics as intrinsic bacterial growth, optimal control simulation, within-host interaction, and multi-scale dynamics, which were not covered in the two earlier reviews [11,12]. As such, it is hoped that the current review could reach a broader scientific community that involves not only epidemiologists and public health professionals, but also applied mathematicians, computational scientists, biologists, immunologists, and other researchers and scholars who are interested in utilizing mathematical models to improve the understanding of cholera dynamics.

This review will focus on mathematical models based on differential equations, including both ordinary differential equations (ODEs) and partial differential equations (PDEs). Such epidemic models are often referred to as compartmental models, which were introduced almost 100 years ago [13] and which still take over the vast majority of modeling studies for infectious diseases, cholera in particular. Starting from an overview of basic cholera transmission models and their applications, model extensions in several directions are surveyed that include spatial and temporal heterogeneities, effects of disease control, impacts of human behavior, and multi-scale infection dynamics. Although the emphasis of this review is the modeling of cholera, the various types of mathematical models and techniques discussed in this article can be applied to many other infectious diseases.

## 2. Basic Transmission Dynamics

The first mathematical model for cholera dynamics was proposed by Capasso and Paveri-Fontana [14], based on two simple equations for the infected individuals and free-living pathogens, to study the 1973 cholera epidemic in the Mediterranean region. Following this seminal work, many mathematical models were proposed for cholera transmission in homogeneous populations and environments. These models typically involve at least four compartments that include the numbers of the susceptible, infected, and recovered individuals, commonly denoted by *S*, *I*, and *R*, respectively, and the concentration of the pathogenic bacterium *V. cholerae* in the aquatic environment, commonly denoted by *B*. More sophisticated models, such as those reviewed in Section 3, Section 4, Section 5 and Section 6, generally build upon such basic transmission dynamics models.

### 2.1. A Few Examples of Cholera Models

A notable extension from the model of Capasso and Paveri-Fontana [14] was made by Codeço in 2001 [15], where the bacterial concentration in the water supply was incorporated into an SIR model to form a combined human-environment epidemiological system. The details of this model are presented in Table 1 and Table 2, with some notations slightly different from those in the original formulation of [15]. The incidence, which determines the rate of new infection, is represented by a Michaelis-Menten type functional response [16], where the half saturation rate κ refers to the infectious dose in water sufficient to produce infection in 50% of those exposed. The existence of such a dose-response relation is supported by experimental evidence that the frequency and severity of cholera infection were correlated with inoculum [17]. Only the environment-to-human transmission pathway is considered in this model, which is also referred to as the indirect transmission route, in contrast to the more familiar human-to-human (or, direct) transmission route. Disease-induced mortality is not included in this model (and most other cholera transmission models), given that the case fatality rate for cholera is lower than 1% in general [18]. However, a cholera model can be easily modified to account for disease-induced deaths in a particular epidemic scenario.

Using a nonlinear incidence similar to that in Codeço’s model, Hartley et al. [19] in 2006 incorporated a hyper-infectious stage of *V. cholerae* and developed a new cholera transmission model. They introduced two pathogen compartments, $B_{L}$ and $B_{H}$, to denote the lower and hyper infective stages of *V. cholerae*, respectively (see Table 1 and Table 2). The emphasis of the “explosive” infectivity of *V. cholerae* was based on laboratory measurements that freshly shed *V. cholerae* from human intestines outcompeted other *V. cholerae* by as much as 700-fold for the first few hours in the environment [20,21].

Mukandavire et al. [22] proposed a model with both the indirect and direct transmission routes to estimate the reproduction number for the 2008–2009 cholera outbreak in Zimbabwe. The incidence in this model consists of two parts: one is the environment-to-human transmission which is again represented by a Michaelis-Menten functional response form, and the other is the human-to-human transmission which is represented by a standard bilinear form (see Table 1 and Table 2).

The basic reproduction number, commonly denoted by $R_{0}$, is frequently used in epidemic studies to measure the infection risk. It is defined as the average number of secondary infections that occur when one infective is introduced into a completely susceptible host population [10,23]. The next-generation matrix technique described in [24] is a standard mathematical approach to compute the basic reproduction number $R_{0}$. With this approach, the basic reproduction number for each of the three cholera models can be derived, and the results are listed in Table 1. For example, $R_{0}=N\xi \beta /\kappa \delta (\gamma +\mu )$ for the model of Codeço [15], where the term ξβ/κδ represents the (normalized) unit transmission rate from the environmental pathogen to the human host, 1/(γ+μ) represents the expected time of infection, and the multiplication of these two terms with the human population size *N* gives the expected number of secondary infections during one generation period. The basic reproduction number for the model of Hartley et al. [19] consists of two parts that represent, respectively, the contributions from the hyper-infective and lower-infective vibrios. Meanwhile, the basic reproduction number for the model of Mukandavire et al. [22] is shaped by both the environment-to-human and human-to-human transmission pathways.

**Table 1:** Examples of basic cholera transmission models: Codeço [15], Hartley et al. [19], and Mukandavire et al. [22]. The prime symbol ${}^{\prime }$ denotes the derivative with respect to time.

Model	Mathematical Formulation	Basic Reproduction Number
[15]	$\begin{array}{cc} S^{\prime } & =\mu N-\beta SB/(\kappa +B)-\mu S\\ I^{\prime } & =\beta SB/(\kappa +B)-(\gamma +\mu )I\\ R^{\prime } & =\gamma I-\mu R\\ B^{\prime } & =\xi I-\delta B \end{array}$	$\frac{N\xi \beta }{\kappa \delta (\gamma +\mu )}$
[19]	$\begin{array}{cc} S^{\prime } & =\mu N-\beta_{L}SB_{L}/\left(\kappa_{L}+B_{L}\right)-\beta_{H}SB_{H}/\left(\kappa_{H}+B_{H}\right)-\mu S\\ I^{\prime } & =\beta_{L}SB_{L}/\left(\kappa_{L}+B_{L}\right)+\beta_{H}SB_{H}/\left(\kappa_{H}+B_{H}\right)-\left(\gamma +\mu \right)I\\ R^{\prime } & =\gamma I-\mu R\\ B_{H}^{\prime } & =\xi I-\chi B_{H}\\ B_{L}^{\prime } & =\chi B_{H}-\delta B_{L} \end{array}$	$\frac{N\xi }{\gamma +\mu }\left(\frac{\beta_{H}}{\kappa_{H}\chi }+\frac{\beta_{L}}{\kappa_{L}\delta }\right)$
[22]	$\begin{array}{cc} S^{\prime } & =\mu N-\beta_{e}SB\left(\kappa +B\right)-\beta_{h}SI-\mu S\\ I^{\prime } & =\beta_{e}SB/\left(\kappa +B\right)+\beta_{h}SI-\left(\gamma +\mu \right)I\\ R^{\prime } & =\gamma I-\mu R\\ B^{\prime } & =\xi I-\delta B \end{array}$	$\frac{N}{\kappa \delta (\gamma +\mu )}\left(\xi \beta_{e}+\kappa \delta \beta_{h}\right)$

**Table 2:** Parameters for the basic cholera transmission models presented in Table 1. For the models of Hartley et al. [19] and Mukandavire et al. [22], only those parameters that do not appear in the model of Codeço [15] are listed.

Model	Parameter & Definition
[15]	*N* Human population size μ Natural birth and death rate for humans β Contact rate with *V. cholerae* in the environment κ Half saturation rate for *V. cholerae* γ Recovery rate for infected people ξ Rate of contribution from an infected person to *V. cholerae* in the environment δ Removal rate of *V. cholerae* in the environment
[19]	$\beta_{L}$ Contact rate with lower-infectious *V. cholerae* in the environment $\kappa_{L}$ Half saturation rate for lower-infectious *V. cholerae* $\beta_{H}$ Contact rate with hyper-infectious *V. cholerae* in the environment $\kappa_{H}$ Half saturation rate for hyper-infectious *V. cholerae* χ Rate of decay for *V. cholerae* from hyper-infectivity to lower-infectivity
[22]	$\beta_{e}$ Contact rate with *V. cholerae* from the environment-to-human pathway $\beta_{h}$ Contact rate with *V. cholerae* from the human-to-human pathway

There are quite a few other mathematical models developed for cholera transmission in a homogeneous population. For example, Tien and Earn [25] proposed a waterborne infection model that also includes dual (environment-to-human and human-to-human) transmission pathways. A bilinear incidence was employed for each transmission route, and no saturation effect was considered in this work. Jensen et al. [26] published a model with an emphasis on how lytic bacteriophage specific for *V. cholerae* impacts cholera outbreaks. A strongly nonlinear incidence form was utilized in this model, and a nonlinear growth for *V. cholerae* in the environment was considered. A more general modeling framework was developed in [27], which allows various representations for the force of infection resulting from environment-human and human–human interactions, and for the bacterial dynamics in the environment. Many published cholera models (such as those in [15,19,22,25,28]) can be included in this framework as special cases.

Most of these cholera transmission models employ the standard compartments of the susceptible (*S*), the infected (*I*), and the recovered (*R*) to describe the flow of infection in a human population. The underlying assumption is that an individual infected with cholera becomes immediately infectious. In reality, there is typically a short incubation period for cholera infection. A systematic review [29] estimated that the median incubation period of cholera was 1.4 days. Mathematical models can incorporate the impact of the incubation period by adding an exposed (*E*) compartment, and such models can possibly improve the accuracy of predictions for the transmission and spread of cholera [30,31]. In addition, we refer to [32] for detailed and insightful discussion regarding potential issues such as model misspecification and parameter uncertainty related to some basic cholera models.

### 2.2. Transmission Routes

Most of the earlier cholera models (e.g., [15,19,26,28,33]) considered only the indirect, environment-to-human transmission route. In particular, the model of Hartley et al. [19] introduced a hyper-infectious stage of *V. cholerae* to represent the freshly shed pathogen, which was well supported by experimental findings [20]. As pointed out by Pascual et al. [34]; however, the role of the hyper-infectious stage of *V. cholerae* implicitly highlights the importance of human–human interaction in a short time frame following the onset of symptoms (typically within 24 h). This is especially relevant for the households of infected individuals, where high secondary attack rates are often observed [35]. For example, the hands of an infected individual may be contaminated by freshly shed vibrios. When this person uses dirty hands to contact other people (shaking hands, hugging, etc.) or to prepare food for members of the household, the infection can be easily transmitted due to the high infectivity of the vibrios at this stage. In this regard, the indirect incidence term based on the hyper-infective bacterial stage in the model of Hartley et al. may be represented, perhaps equivalently, by the human-to-human transmission pathway that corresponds to the direct incidence term in the model of Mukandavire et al. [22] as well as those in other models [25,27,36].

These two modeling perspectives, i.e., the incorporation of the hyper-infectious stage of *V. cholerae* through the environment-to-human transmission route and the use of the human-to-human transmission pathway, have both been well known to the cholera-modeling community in recent years. In general, it may be difficult to assess which one is better than the other. The choice between these two modeling approaches appear to mostly depend on the specific purpose of a cholera model and possible invention methods considered there. We refer to the reviews [11,12] for additional discussion regarding this point.

### 2.3. Intrinsic Bacterial Dynamics

The majority of the basic cholera transmission models employed a simple representation of the bacterial dynamics in the environment; see, e.g., the equation for *B* in the models listed in Table 1. Two linear terms were typically involved to describe the rate of change for the environmental vibrios, with the positive term representing the contribution from infected people through shedding, and the negative term representing the natural removal of the bacteria. The underlying assumption, related to an early theory in cholera ecology [37], was that the vibrios would not be able to sustain themselves in the environment without the human contribution (e.g., shedding from infected individuals and inflow from contaminated sewage). This assumption allowed a simplification in the development, analysis, and implementation of these cholera models. However, even with such simplified models, it was found that there was large uncertainty associated with the parameterization of the bacterial dynamics, particular for the lifespan of *V. cholerae* in the water supply [12,32]. In fact, recent ecological studies [38,39,40,41] have provided strong evidence that *V. cholerae* can independently survive and multiply in various aquatic environments, including freshwater, estuaries, and seawater.

**Figure 2:**
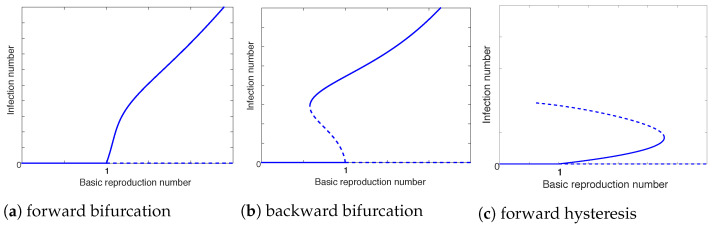
Three different types of bifurcation behaviors: (**a**) forward bifurcation; (**b**) backward bifurcation; (**c**) forward hysteresis. Solid and dashed lines represent stable and unstable equilibria, respectively. The horizontal axes represent the disease-free equilibria, and the lines above the horizontal axes represent the endemic equilibria.

Several mathematical models have incorporated nontrivial, intrinsic bacterial dynamics into the study of cholera transmission [26,27,42,43]. In particular, an analysis was conducted in [43] for two types of nonlinear bacterial dynamics: one for logistic growth, and the other for cubic growth with possible Allee effects. The logistic growth model follows regular threshold dynamics, similar to those observed from previous cholera models based on linear bacterial dynamics: when $R_{0}<1$, the disease will be eradicated, characterized by a stable disease-free equilibrium; when $R_{0}>1$, the disease will persist and become endemic, characterized by the instability of the disease-free equilibrium and the appearance of a stable endemic equilibrium. Mathematically, this threshold behavior is known as a forward bifurcation, depicted in Figure 2a. In contrast, the model with Allee effects exhibits very rich dynamics, including the existence of multiple endemic states when $R_{0}<1$, as illustrated in Figure 2b, and when $R_{0}>1$, as illustrated in Figure 2c. These two types of dynamical behaviors are referred to as backward bifurcation and forward hysteresis, respectively. Unlike the forward bifurcation, where a reduction of $R_{0}$ below unity would lead to disease eradication, these two scenarios indicate potential challenges in the control of cholera outbreaks. For example, when a backward bifurcation occurs, there exist both stable and unstable branches of positive endemic equilibria, an indicator for disease persistence, in the region $R_{0}<1$. Hence, simply reducing $R_{0}$ below unity would not be sufficient to eliminate the infection, and stronger control measures have to be implemented (so that $R_{0}$ can be pushed to the small region free of positive equilibria) to contain the epidemic and eradicate the disease. More discussion for the backward bifurcation related to cholera dynamics can be found in [44,45]. Additionally, a forward hysteresis is often accompanied by a backward bifurcation, though not shown in Figure 2c, which could lead to a potentially catastrophic epidemic characterized by a rapid increase from low prevalence to high prevalence.

Although not as extensively discussed as for animal populations, Allee effects in populations of microorganisms such as bacteria have been reported in several studies [46,47,48,49]. Thus, the theoretical study conducted in [43] could be practically relevant in terms of cholera prevention and intervention, especially for control measures (such as water sanitation) that target the reduction and removal of *V. cholerae* in the environment. Further development of mathematical models for cholera dynamics along this direction would benefit from biological and ecological studies focused on detailed growth patterns of *V. cholerae* under various environmental conditions.

### 2.4. Real-World Applications

A number of modeling studies have been conducted for real-world cholera outbreaks using data reported by government agencies and public health administrations. Many of these studies utilized relatively simple cholera models that belong to the basic transmission models discussed earlier in this article.

Most of these application studies were concerned with the Haiti cholera outbreak during 2010–2012. For example, Abrams et al. [50] conducted real-time modeling for the Haiti cholera outbreak and projected the cases and hospitalizations during the first year of the outbreak based on available surveillance data. Their model included two categories of recovered individuals that represent the effect of waning immunity. Andrews and Basu [51] constructed a mathematical model that considered environmental reservoirs for both hyper-infectious and lower-infectious vibrios and included both symptomatic and asymptomatic infections. The model was then calibrated using hospitalization and mortality data reported by the Haitian Ministry of Health. Chao et al. [30] developed a transmission model to assess different vaccination strategies for epidemic cholera in Haiti. Tuite et al. [52] applied a model with both direct and indirect transmission pathways to investigate the infection risk of each administrative department in Haiti during the cholera outbreak, and generated reproduction numbers ranging from 2.06 to 2.78 for different regions of Haiti. Eisenberg et al. [53] modeled the relationship between rainfall and the Haiti cholera outbreak, and found that increased rainfall was associated with increased cholera risk. Some other modeling studies for the Haiti cholera outbreak include [54,55,56,57,58,59,60,61].

In addition, modeling studies for the 2008–2009 Zimbabwe cholera outbreak include [22,62,63], and those for the Yemen cholera outbreak starting from 2016 include [64,65,66,67]. Modeling studies for cholera outbreaks in other countries and regions include, for example, [68,69,70,71].

## 3. Spatial and Temporal Heterogeneities

### 3.1. Multi-Group and Multi-Patch Models

Transmission of cholera, like that of many other infectious diseases, is complicated by spatial heterogeneity that involves different ecological and geographical environments, population sizes, mobility and contact patterns, and socio-economic and demographic structures.

Mukandavire et al. [22] performed a modeling study for the 2008–2009 Zimbabwe cholera outbreak, where basic reproduction numbers were estimated and relative contributions from direct and indirect transmission routes were compared for the 10 provinces in Zimbabwe. The results were highly heterogeneous, an indication that the underlying transmission pattern varied substantially throughout the country. Similarly, the study in [52] generated a range of reproduction numbers for different administrative departments in Haiti during the 2010 cholera outbreak. In addition, an investigation of the Yemen cholera outbreak during 2016–2017 [64] revealed that the transmission modes and infection risk differ significantly in the northwest, southwest, and east regions of the country. Although relatively simple mathematical models were used in these studies, the findings confirmed that spatial heterogeneity plays an important role in cholera transmission and spread. Consequently, there is a need for more detailed quantitative investigation regarding the spatial effects, especially the movement of human hosts and the dispersal of pathogenic vibrios, on cholera epidemics and endemicity.

Meta-population models [72,73] have been commonly used in epidemiological studies to incorporate spatial heterogeneity from the hosts and environments. A standard approach is based on multi-group modeling [74,75,76], where the entire population is divided into a number of groups that possess different characteristics. Each group is connected to other groups, and infection can take place between individuals within the same group or from different groups. The multi-group formulation is analogous to the Lagrangian approach in fluid dynamics since it labels individual hosts of different groups and explicitly tracks disease transmission for individuals [77].

A multi-group cholera model was proposed and analyzed in [78] which considered only the indirect, environment-to-human transmission route. The authors in [79] extended the homogeneous cholera model presented in [27] to a multi-group setting, and found that the overall infection risk for the entire population represents a combination of the transmission risk for each individual group. Another multi-group model, applicable to cholera transmission, was proposed in [80] with both direction and indirect transmission pathways represented in a general incidence form. Other cholera-modeling studies based on the multi-group framework include, for example, [52,81,82].

Another popular meta-population approach, called multi-patch modeling [83,84,85,86], divides the entire population into a set of patches, where each patch is often associated with a different location. This method describes the movement of the hosts and/or pathogens between patches, with a focus on the pathogen transmission within each patch. This type of formulation is related to the Eulerian approach in fluid dynamics as it labels locations and explicitly tracks disease transmission for each location.

A multi-patch cholera model was developed in [87] where the movement of the pathogenic bacteria between different patches was considered and only the indirect transmission route was included. This modeling framework was subsequently extended to predict the spatial evolution of the Haiti cholera outbreak [54,55]. Another modeling study for Haiti cholera outbreak was performed in [52], where the between-patch epidemic spread was based on a gravity model that depends on the population size and distance between regional centroids. In another multi-patch model [88], the movement of both the human hosts and environmental vibrios was incorporated, and both the direct and indirect transmission pathways were included. A sharp threshold condition was established at $R_{0}=1$ for the entire system to distinguish disease extinction ($R_{0}<1$) and disease persistence ($R_{0}>1$). A cholera model that couples the multi-patch structure with a time-periodic environment was proposed and analyzed in [89]. Some other cholera models were proposed in [90,91] where the human hosts from different patches do not directly communicate with each other and, instead, are connected through a common environmental water reservoir. Their model structure can be regarded as a star network where the hub (or, center) corresponds to the shared water source and the leaves (or, vertices) correspond to the host patches. The basic reproduction number is determined by the direct transmission in each patch and the total indirect transmission through the water source from all patches.

In most of these studies, it was found that the basic reproduction number and the outbreak size would be higher for the coupled meta-population system than those for the disconnected, individual groups or patches, indicating that increased spatial heterogeneity may lead to increased disease transmission risk. In some cases, it was also found that the connection between population groups or patches would allow cholera to persist, whereas such disease persistence may not be possible in any isolated individual population [90].

### 3.2. Reaction-Diffusion PDE Models

Partial differential equations (PDEs) of the reaction–diffusion type are extensively used in epidemiological modeling (e.g., [92,93,94,95,96,97]). Fick’s law [98] can be generally applied to construct a reaction–diffusion model. Often, based on an epidemic system of ordinary differential equations (ODEs), diffusion terms can be added to model the spatial spread of the disease. A diffusion process represents random movement and dispersal of hosts and/or pathogens over a spatial domain, normally without a directional preference. The underlying ODE model typically describes homogeneous dynamics of disease transmission, whereas the reaction–diffusion PDE model incorporates spatial movement, generally associated with location-dependent diffusion rates, into the epidemiological process and emphasizes the spatial heterogeneity of population dynamics [99,100] related to disease transmission and spread.

A reaction–diffusion model, derived from the continuous limit of a multi-patch ODE system, was presented in [87] to account for the epidemic spreading of cholera. The spatial dispersal of *V. cholerae* was modeled as a diffusion process, and only the environment-to-human transmission route was considered. This model was extended in [101] to include the movement of human hosts. Another cholera model was developed in [102] where the human hosts undergo a diffusion process while the vibrios remain stationary. In [42], a PDE cholera model was proposed that represents the spatial diffusion of both the pathogens and human hosts, while incorporating both the direct and indirect transmission routes. This work was later extended in [103] to include a convection process for the pathogenic bacteria, such as the movement of the vibrios from the upstream to the downstream along a river. These cholera models and some other extensions were mathematically analyzed in a rigorous way in [104,105,106]. Additionally, the work in [107] incorporated seasonal fluctuation into the spatiotemporal dynamics of cholera.

For all the aforementioned PDE-based cholera studies, the spatial domain is restricted to either a one-dimensional (1D) space or a symmetric two-dimensional (2D) space that is equivalent to a 1D domain. These simplified, 1D reaction–diffusion models may be practically meaningful when the spread of cholera is associated with a fluvial system. For example, the suspected source of the 2010–2012 Haiti cholera outbreak outbreak was Artibonite River, the longest and most important river in Haiti, and the initial spread of the disease was believed to follow the river [108].

More sophisticated PDE models of the reaction–diffusion type that involve multi-dimensional spatial domains have also been developed for cholera dynamics; see, e.g., [109,110,111,112]. These modeling studies have focused on the mathematical analysis of the PDE systems.

All these PDE studies contribute to the body of knowledge in mathematical modeling of cholera. On the other hand, most of these studies are intentionally theoretical, and it remains a challenge to apply such reaction–diffusion models to fit data from real-world cholera outbreaks. Particularly, the diffusion coefficients, which generally take different values for different population groups and spatial locations, are difficult to calibrate. Thus far, there is very little published work regarding the outbreak simulation and practical data fitting of reaction–diffusion cholera models, even for the simplified cases with 1D spatial domains and constant diffusion coefficients. The challenge associated with reaction–diffusion modeling is not only for cholera, but also for many other infectious diseases. More efforts along this direction are needed to facilitate the real-world applications of these PDE epidemic models and to make such models better appreciated by the public health community.

### 3.3. Seasonal Variation and Climate Change

The transmission of cholera is inherently related to the environment. Many environmental factors, such as floods, droughts, precipitations, and water temperature and salinity, are seasonal and can significantly impact cholera dynamics [113,114,115,116]. For example, it has been observed that cholera becomes a seasonal disease in many endemic places and infection peaks typically occur in the rainy or monsoon season on an annual basis [117,118]. Furthermore, historical cholera data indicate that climate change, which leads to rises in sea levels and global temperatures, may influence the temporal fluctuations of cholera and increase the frequency and duration of cholera outbreaks [119,120].

Most cholera models based on ODE systems utilize constant parameters for simplicity, and these models may not be able to reflect the seasonal and climatic behavior of cholera dynamics. To overcome this difficulty, non-autonomous ODE systems with time-dependent parameters can be used. In particular, temporal periodicity may be applied to the contact rate, recovery rate, and pathogen growth rate, among other parameters, to represent regular seasonal oscillations of the infection dynamics.

Simple numerical tests were conducted in [15] for three hypothetic scenarios with periodic model parameters. A more general cholera model [121] incorporated periodicity into both the incidence and pathogen functions to represent seasonal oscillations in a generic manner. This model was extended in [122] to a stochastic system based on a Markov process, where it was shown that the probability of a cholera outbreak is periodic in time. Another study [36] discussed the intra-annual seasonality and variability of cholera dynamics based on a mathematical model that incorporates both asymptomatic and symptomatic infections. The authors of [123] studied the seasonality of cholera dynamics and the fluctuations of the aquatic reservoir in endemic areas driven by rainfall and temperature, and fitted their model to the historical cholera dataset of the Bengal region in the Indian subcontinent. Another cholera study [124] incorporated seasonal environmental drivers, including river flow, temperature and chlorophyll concentration, into a spatially explicit model and showed that such drivers may generate dual-peak cholera prevalence patterns. A mathematical model presented in [125] showed that climate variability played a vital role in modulating the size of cholera outbreaks in Bangladesh. In addition, the authors in [126] reviewed several mathematical models and provided quantitative evidence for the influence of climate change on cholera dynamics.

In general, the seasonal patterns and temporal variations of cholera epidemics and endemicity are complex, involving the interplay between many environmental and climatic factors. Mathematical models based on periodic systems (i.e., systems of differential equations with time-periodic parameters) are capable of simulating and predicting regular seasonal oscillations of cholera outbreaks, but these may not represent the full picture of the intra- and inter-annual dynamics of cholera. Particularly, the effects of climate change are typically not periodic and thus cannot be resolved through purely periodic models. Instead, the use of time-dependent but non-periodic model parameters would be more appropriate in this case, though the models may become difficult to analyze and may involve non-trivial data fitting procedures to calibrate the parameters. Furthermore, as pointed out in [123], stochasticity played an important role in the occurrence of some abnormally large cholera outbreaks in the Bengal region, while regularity of inter-annual cholera dynamics was found in other times (with periodicity roughly corresponding to the dominant frequency of El Niño). This indicates that a combination of periodicity and stochasticity into a single modeling framework may better explain the various environmental and climatic drivers and provide deeper insight into the temporal dynamics of cholera.

## 4. Effects of Disease Control

The frequent occurrence of cholera outbreaks and their increasing duration and severity underscore the importance of effective cholera control. Common prevention and intervention methods for cholera include the rehydration therapy, antibiotic treatment, water sanitation, and vaccination. Oral rehydration solution with a mix of salt, sugar, and clean water is widely used to treat individuals with minor or moderate infections. This basic therapy has been credited for preventing tens of millions of deaths since they were formally endorsed by WHO and for reducing the average cholera case fatality rates below 1% [5,18]. Antibiotics such as doxycycline, ciprofloxacin, and azithromycin are recommended for severe cases where hospitalization is typically required. Antibiotic therapy may reduce the duration of symptoms, the volume of diarrhea, and the length of time that the vibrios are excreted in the feces. A significant concern, however, is that antibiotic therapy frequently leads to antimicrobial resistance, which may complicate the treatment of cholera and may even result in higher rates of secondary infection [127]. Water sanitation based on chlorination, filtering and other cleaning/disinfecting methods is an effective way to improve the quality of drinking water and to reduce the concentration of the pathogens in the environment, which is crucial for the prevention of cholera and other waterborne infections in the long run. The impact of this approach, though, may be limited in an emergency setting associated with a disease outbreak. In addition, with the introduction of low-cost oral vaccines based on live attenuated or killed whole-cells, vaccination has been an effective and affordable means to fight cholera, with a series of trials and campaigns successfully completed in various endemic places during the last few decades [127,128]. Vaccination in epidemic and emergency situations was also conditionally recommended by WHO [129], and was successfully implemented during the 2010–2012 Haiti cholera outbreak [130].

A number of mathematical models have been published to quantify the effects of these control measures on the transmission and spread of cholera. For example, a system of differential equations was constructed in [131] that incorporated antibiotic treatment, vaccination and water sanitation into cholera transmission dynamics. An analysis for cost-effective strategies to curb cholera transmission in epidemic settings was also conducted. Similarly, the work in [132] included vaccination and water disinfection in a cholera transmission model. The cholera model proposed in [22] was applied to each of the 10 provinces in Zimbabwe to estimate the minimal vaccination coverage required to contain the 2008–2009 cholera outbreak. The studies in [30,51] designed mathematical models to simulate the epidemic trajectories for the Haiti cholera outbreak in 2010 and estimate the impact of clean water, vaccination and enhanced antibiotic distribution programs. In [133,134], the authors considered the effects of quarantine on the transmission dynamics of cholera. Another modeling study was conducted in [135] that incorporated the impact of disease education programs into the transmission rates and the effects of water sanitation into the environmental pathogen dynamics. Additionally, the models proposed in [136,137] incorporated age-structures and were focused on the impact of vaccination, with a rigorous mathematical analysis.

Practically, public health resources are limited, and not all the disease control strategies are feasible in a specific location within a certain timeframe. Meanwhile, the implementation of any control measures would incur expenses. An optimal control study [138], which takes into account the costs of cholera control, seeks a cost-effective solution to manage a cholera outbreak. The results could provide useful guidelines for effectively containing a cholera outbreak while reducing the total costs of the disease management.

Most optimal control studies for cholera employ ODE models, based on Pontryagin’s Maximum/Minimum Principle [139] and other standard optimal control theories [140]. A general procedure for such a study starts with a clearly defined goal of optimal control, mathematically represented by an objective functional, which is often formulated in such a way as to minimize the number of infected individuals and the costs of disease control measures in a prescribed time interval. A Hamiltonian is then constructed using the adjoint variables, which are associated with those state variables such as *S*, *I*, *R* and *B*, and the objective functional. The problem of minimizing the objective functional is then transformed into a problem of minimizing the Hamiltonian with respect to the control. From there, one can normally derive the adjoint system (with final-time conditions) and the characterization of the control variables. Together with the original epidemic system (referred to as the state system), they constitute a complete optimal control model. A popular numerical approach to solve such a coupled, nonlinear control system is the forward-backward sweep method [138], which involves an iterative process—at each iteration, the state system is solved forward in time, then the adjoint system is solved backward in time, and then the control variables are updated. Numerous optimal control studies for cholera are based on these analytical and numerical techniques; see, e.g., [33,66,131,135,141,142,143,144]. Meanwhile, some extensions to the optimal control of stochastic cholera models have been made [145,146].

A few optimal control studies based on PDE models, especially age-structured PDE systems, have also been published for cholera transmission dynamics [137,147,148,149]. These studies typically involve deeper mathematical theory in developing the optimal control models and require more computational efforts in finding the solutions.

Mathematical models incorporating cholera control can potentially better describe the transmission and spread of cholera at the present age. In large cholera outbreaks, some control measures are always implemented that would impact the transmission dynamics and progression trajectory of cholera. The modeling studies can quantify and compare different prevention and intervention strategies and evaluate their outcomes. Furthermore, an optimal control study can theoretically predict which control strategy, or a combination of several strategies, could achieve the best performance in balancing the effects and costs of cholera outbreak management.

On the other hand, there are still several challenges in the practical applications of these cholera control models toward policy development. For example, the parameters associated with the interventions, such as the therapeutic treatment rate, vaccination rate, and water sanitation rate, are generally time-dependent and accurate estimates of these parameter values may be challenging. Meanwhile, data that link the strength of control measures and the reduction of disease transmission are not always available, which may hinder the quantification of the effects of disease control. For another example, an optimal control study typically needs to represent the reduction of the prevalence and/or mortality in terms of some monetary value, and such a value may be difficult or even impossible to assess in a practical situation. Furthermore, some of these cholera control studies should be understood as hypothetical, and their potential of solving real-world problems has yet to be utilized.

## 5. Impacts of Human Behavior

There is usually a two-way interaction between an epidemic and human behavior. A disease outbreak would likely raise the awareness of the infection risk that often leads to changes of population activity and social behavior. In return, behavioral changes could play an important role in shaping the transmission pattern and epidemic progression [150]. When the prevalence level is high, at least some people would attempt to adjust their routine schedules in work and travel and take necessary action to reduce contacts with infected individuals so as to protect themselves and their families. They would also be motivated to make changes in their social activities, such as practicing self-quarantine, wearing masks, and adhering to social distancing, which would help to slow down the transmission and spread of the infection.

Particularly, when cholera is concerned, people who are conscious of the infection risk would possibly pay more attention to the hygiene and sanitation practice, such as washing hands often with soap, properly treating disposals, and boiling or disinfecting water before drinking. These people may also be willing to receive vaccination, and would likely avoid contacts with infected individuals and contaminated water or food [151]. Currently, the communication regarding an outbreak has gone far beyond the traditional sources such as newspaper and television and radio stations. The advance of information technology allows fast and up-to-date case reports from the internet and mobile networks, including various social media and search engine sites, which could promptly motivate behavioral changes from the general public in order to reduce the risk of infection. Consequently, the disease transmission rate during an epidemic, cholera in particular, is typically time-dependent and often inversely correlated to the disease prevalence. Hence, positive changes of human behavior could contribute significantly to the control and possible eradication of the disease [152,153].

There are an increasing number of epidemic modeling studies concerned with human behavior (see, e.g., [154,155,156,157] and references therein). Specifically, several mathematical models have focused on human behavior related to the transmission and spread of cholera. In a cholera model proposed in [158], the impact of human behavior was incorporated into the direct and indirect transmissions rates as well as the pathogen shedding rate from the infectious hosts. The results showed that positive changes of human behavior can reduce the risk of infection, decrease the epidemic size and endemic level, and reduce the spatial spreading speeds of cholera. The authors in [159] proposed two models to study human behavior associated with disease awareness programs and its impact on cholera transmission dynamics, and found that these two models exhibit significantly different dynamical properties. Their findings highlighted the importance of validating key assumptions in the selection and implementation of practically meaningful cholera models. The cholera model in [160] compared the effects of disease education and water chlorination, and found that education is more effective than chlorination in decreasing bacterial concentrations and reducing the number of cholera cases. Additional modeling studies for human behavior and cholera dynamics include, among others [135,161,162].

Although these studies have generated very useful insight for the epidemiological impact of human behavior, most of the findings remain theoretical with what-if scenarios and have not been validated by real behavioral data or applied for decision making. Meanwhile, there is currently an insufficient representation of the complex nature of human behavior, which involves many different angles from social interaction, behavioral characteristics, psychological effects, economic concerns, and population heterogeneity. In particular, responses to interventions could vary significantly both within and between populations, depending on factors such as cultural and religious circumstances, perceived infection risk, and health campaign coverage. How to accurately translate such complex behavioral dynamics into disease modeling remains an open question. A deeper investigation in this direction would require an integration of the behavioral, social, and economic processes into next-generation epidemic models. Once developed, such models can potentially yield a better understanding and more reliable prediction for the spread and progression of cholera and many other infections. To that end, collaborations between mathematical modelers, epidemiologists, and social and behavioral scientists would be essential.

## 6. Multi-Scale Dynamics

### 6.1. Within-Host Modeling

In contrast to the large number of published mathematical models that are concerned with the transmission and spread of cholera at the population level, relatively few modeling studies have been devoted to the bacterial dynamics of cholera inside the human body and the related host–pathogen interaction.

Experimental findings indicate that cholera infection involves unique and complicated within-host dynamics [163]. In particular, a virus, referred to as the cholera toxin phage (CTXϕ), plays a critical role in the pathogenesis of the bacterium *V. cholerae* inside the human body. CTXϕ is originally present as an integrated section of the genome of *V. cholerae*, and it remains silent within *V. cholerae* in the natural aquatic environment. However, after the vibrios enter the human body and reach the intestinal area, the viral particles within some vibrios are activated. Through a horizontal gene transfer, the virus transduces the vibrios ingested from the environment into another type of vibrios that have an infectivity up to 700-fold increase [1,19,39]. The released virus may enter other vibrios, and such bacterial–viral interaction leads to the production of a large amount of toxin causing severe diarrhea.

A model for the within-host dynamics of cholera was proposed in [164] to describe the bacterial–viral interaction that leads to the transformation of the vibrios ingested from the environment (with lower infectivity) to hyper-infectious vibrios inside the human body. A saturation-based response form was used to represent the within-host interaction. This model was subsequently extended in [165] to include the innate immune response in the bacterial–viral–immune interaction. It was found that the basic reproduction number of the extended model is given by $R_{0}=\max\left\{R_{1},R_{2},R_{3}\right\}$, where $R_{1}$ is the bacterial reproduction number that measures the intrinsic growth rate of the highly infectious vibrios, $R_{2}$ is the viral reproduction number that quantifies the generation rate of CTXϕ, and $R_{3}$ is the immune reproduction number that characterizes the generation rate of the immune cells. The biological interpretation of this result is that the risk of developing cholera infection inside the human body is determined collectively by the bacterial, viral and immune reproduction rates, representing the interplay among these three critical components in the within-host dynamics of cholera. Another within-host cholera model was proposed in [166], where a bilinear incidence was employed to represent the interaction between the bacterium, the virus, and the host immune response.

A limitation of these modeling studies is that only the effect of the innate host immunity is considered, which makes instantaneous responses against pathogens invading the human body. On the other hand, the adaptive immune response also plays an important role in the human immune system. The adaptive immunity kicks in with delayed responses but often leads to more sustained protection of the human body. This process may be mathematically represented by adding a time delay into the differential equations. Meanwhile, many other issues related to the within-host dynamics of cholera, such as the detailed interaction between the virus and the molecular components of the vibrios and immune cells, the spatial structure and heterogeneity associated with different types of cells, and the link between the cellular interaction and the tissue- and organ-level dynamics, have not been addressed in current modeling studies. These could be interesting topics for future model development, and several mathematical techniques for population dynamics, such as multi-patch systems and reaction–diffusion PDEs, may be extended to within-host modeling.

The transmission and spread of cholera depend on the infectiousness of individual hosts, which in turn depends on pathogen load. Thus, the host–pathogen interaction within the human body could have a significant impact on cholera transmission at the population level. Additionally, after the highly infectious vibrios inside the human body are shed out to the environment, they can stay at this hyper-infectious state for a period of several hours, as discussed in Section 2. Individuals who contact such freshly shed vibrios through contaminated water and food or through human–human interaction would more likely be infected with cholera. In this regard, the within-host interaction constitutes an essential part of cholera dynamics which demands further modeling efforts and deeper quantitative investigation.

### 6.2. Coupled Within-Host and Between-Host Modeling

A holistic understanding of the infection dynamics of cholera requires both the between-host transmission and the within-host interaction in a single modeling framework. One complication, however, is that cholera dynamics involve environmental ecology, population epidemiology, microbiology, and immunopathology that span several distinct time scales, with the range from hours to years.

A multi-scale cholera model was first proposed in [167], where a two-way coupling for the within-host and between-host dynamics was established. The results showed that the infection risk of cholera is collectively shaped by the pathogen dynamics inside the human body and the disease transmission at the population level. Meanwhile, by noting that the within-host pathogen dynamics are on a faster scale with a typical range from several hours to a few days, while the between-host disease transmission and spread are on a slower scale with a duration ranging from months to years, an analysis based on separation of time scales was conducted which allowed a more detailed examination into the dynamical behavior at each level. One limitation of this study, however, is that the within-host dynamics sub-model takes a simplistic form of a single differential equation that describes the increased toxicity of the pathogen inside the human body.

Another cholera model [166] coupled the host–pathogen interaction, the between-host transmission, and the environmental evolution of the vibrios at three different scales (fast, intermediate, and slow), respectively. The dynamics at each scale make a contribution to the overall disease risk. The within-host subsystem in this model involved the interaction among the bacterium, the virus, and the host immune response. It was found that the basic reproduction number of this multi-scale model represents the contributions from both the human-to-human and the environment-to-human transmission routes, a result consistent with those from the single-scale cholera transmission models (e.g., [22,25]).

An advantage of the aforementioned multi-scale cholera models is that they provide a mutual (or, two-way) connection between the individual host–pathogen interaction and the population-level disease transmission. The separation of time scales allows considerable simplifications of the models so that it is possible to investigate the infection dynamics at each scale in detail. A disadvantage of these models, however, is that they assume individual hosts have the same internal state; i.e., these studies consider an “average” individual and do not resolve the heterogeneity from person to person.

In [168], a multi-scale model of a different type was proposed for environmentally transmitted diseases, with a particular application to cholera, based on the nested modeling approach [169,170]. In this approach, the within-host and between-host dynamics are coupled via the infection age of individuals. The transmission rates and other between-host parameters explicitly depend on the individual pathogen load and immune strength. Consequently, the disease transmission risk at the population level is dependent on the within-host immunopathology. Although the information flow is unidirectional in the nested model, the age-of-infection structure allows to incorporate the staged progression nature of the disease at the population level, which helps to account for the different infection states among individuals and to examine their impact on the between-host disease transmission and progression.

**Figure 3:**
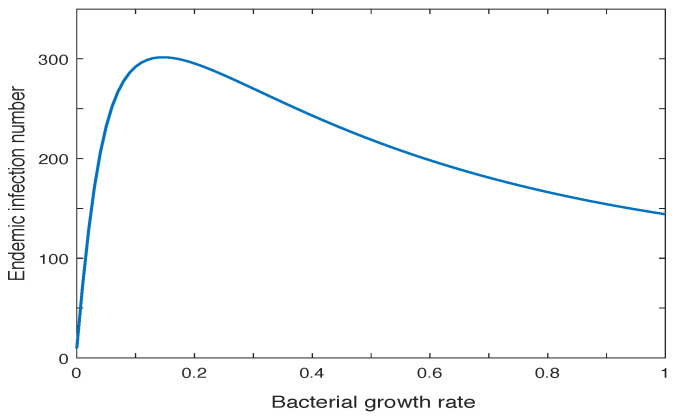
A non-monotone relationship between the infection number at the endemic state and the bacterial growth rate inside the human body.

One interesting result of the modeling study in [168] is that the infection number at the population level does not always monotonically increase with the pathogen load. For example, Figure 3 shows the relationship between the infection number at the endemic state and the bacterial growth rate inside the human body. As the bacterial growth rate increases, the infection number first increases and reaches a peak, and then decreases afterwards. Note that the pathogen load, though varied for different individual hosts, is always positively correlated to the bacterial growth rate. An implication is that a decrease of the pathogen load may not necessarily lead to a reduction of the population-level infection in the long run, unless the bacterial growth rate can be pushed to a small neighborhood of 0 where a positive correlation exists between the two variables.

A limitation of the nested model in [168] is that the between-host dynamics do not have any impact on the within-host dynamics. On the other hand, experimental studies such as [17,163] indicate that the severity of cholera infection for an individual is correlated with inoculum that may depend on different routes of transmission, and that the characteristics of the environmental vibrios could affect the within-host pathogen evolution. Thus far, no cholera models have been able to incorporate the two-way coupling for the between-host and within-host dynamics, while adequately represent the complexity of the host–pathogen interaction and the heterogeneity between individual infection states. This could be a meaningful direction in future efforts of cholera modeling, and development of such multi-scale models can strengthen the connection of cholera dynamics at different scales and enable a more complete understanding of the disease. Additionally, a major challenge at present for the practical application of multi-scale cholera models is the requirement of different types of datasets, including both the population-level epidemiological data and the individual-level immunological data, within the same framework. Hopefully, with the rapid advances of data generation, acquisition and processing in medical science and public health, and with the continuous improvement of multi-scale modeling and fitting techniques, this obstacle will be overcome in the near future.

## 7. Conclusions and Discussion

### 7.1. Complexity of Mathematical Models

A large number of cholera-modeling studies have been published, ranging from very basic models for transmission dynamics in homogeneous populations to highly sophisticated models with spatiotemporal heterogeneities and/or with multiple scales. Those studies based on complex models tend to focus more on the properties of the models, generating qualitative results and contributing to theoretical understanding of the various aspects of cholera dynamics. On the other hand, those studies that have been applied to actual cholera outbreaks are mostly based on simple models, such as the basic transmission models reviewed in Section 2.

Simple models are relatively easy to construct, manipulate, and implement. They typically require only a few parameters to be estimated, which makes it efficient for model calibration with real data. This probably explains why those simple models are more popular in the public heath community, especially for applications to real-world cholera outbreaks. Such practice also applies to the modeling of many other infectious diseases. Another important note is that mathematical models with different levels of complexity might fit the data equally well. For example, as pointed out in [11], a simple SIR model can sometimes produce the dynamics of a cholera outbreak nearly indistinguishable from those models that include the environment-to-human transmission route.

Nevertheless, this does not mean that more sophisticated cholera models should not be used in practical applications. The infection and transmission of cholera involve many spatial and temporal factors at different scales. Basic transmission models may not be able to represent the highly complex nature of cholera dynamics, despite the fact that they may fit the case data well. Data fitting is certainly a critical part of model application, but it is by no means the sole criterion for model selection. When studying a cholera epidemic, several intervention strategies often need to be considered that target different sources and routes of the pathogen. Models should include all the possible transmission pathways in order to compare the intervention methods and to identify the control of which transmission mode would yield the best outcome. When studying cholera in an endemic scenario that involves a large population over a long period of time, different prevention and intervention methods may be needed for distinct subpopulations, or the same control method with varied strengths may be needed for a population group at different times. Models should incorporate the spatial and temporal heterogeneities to manage the disease in a strategic and cost-effective way. In order to study precision medicine for infectious disease control [171], particularly for cholera management, models should connect the within-host and between-host dynamics and investigate the relationship between bacterial evolution, individual pathogen load, and population-level disease transmission in a multi-scale setting. Additionally, multi-purpose and multi-faceted cholera models, with their parameters tunable by different datasets under a variety of scenarios, can be very helpful to researchers and administrators in public heath.

Practical implementation of complex models often requires nontrivial, possibly innovative, data fitting techniques. Model validation becomes especially important and should always be an indispensable part in model development and application. Identifiability analysis [172,173] and sensitivity analysis [174] should be conducted to better understand the properties of the parameters when applying a model to real data. Advanced numerical methods may also be needed to handle complex models, such as those based on strongly nonlinear PDEs or multi-scale ODEs. Fortunately, the rapid growth in computing power allows the implementation and application of highly complex infectious disease models through computationally intensive approaches using large-scale simulation [175,176]. Meanwhile, the continuous improvement in the quantity and quality of epidemic data, especially the increased granularity of surveillance data in time and space, and the availability of high-resolution data from many related fields such as ecology, immunology, and social science, would promote the real-world applications of complex models.

### 7.2. Other Modeling Studies for Cholera

Due to the large volume of the published work in this research area, it is not possible to survey all the cholera models and related mathematical techniques. Some differential equation-based modeling categories not reviewed in this article include, but are not limited to, age-structured cholera models [136,177,178], multi-stage cholera models [179,180], multi-strain cholera models [181,182], stochastic cholera models [122,183,184,185], cholera models with time delay [186,187,188], and cholera models with Hopf bifurcation [161,189,190]. Interested readers may refer to these studies and references therein.

In addition to differential equations, several other types of modeling techniques have been applied to the transmission and spread of cholera. For example, agent-based modeling was conducted in [191,192], discrete-time models were developed in [193,194], machine learning methods were utilized in [195,196,197,198,199], and statistical analysis was applied in [200,201]. These methods complement the standard epidemic modeling approach based on systems of differential equations and enrich the modeling studies for cholera dynamics.

### 7.3. Future Perspectives

Despite a myriad of clinical and theoretical studies and tremendous investment in public health management, cholera is persistent and remains a significant burden in many countries and regions throughout the world. Mathematical models can improve our qualitative and quantitative understanding of cholera, thus contributing to the efforts toward filling the gap between our current knowledge and the complex mechanisms involved in the infection, transmission, spread, and control of cholera.

Remarkable progress has been made in the mathematical modeling of cholera over the last few decades. However, there are still a number of challenges that remain in this research area, including, but not limited to, prediction of possible occurrence of the next large outbreak, forecasting of epidemic progression beyond a short term, quantification of intertwined seasonal and climatic impacts on disease endemicity, characterization of contact structure and movement patterns in heterogeneous populations, representation of complex human behavior in social networks, cost-effective health management strategies in the presence of spatiotemporal heterogeneities, efficient disease control spanning multiple temporal and spatial scales, and precision medicine in the context of a large population. Some of these modeling challenges apply not only to cholera, but also to a wide range of infectious diseases.

To address these challenges and to advance the modeling studies for cholera, traditional mechanistic models, such as those based on differential equations, will benefit from new insights introduced by other emerging modeling techniques. In particular, with the exponential growth of data in recent years, machine learning methods have become increasingly popular in epidemiology [202], and they offer another powerful tool to study cholera dynamics from a data-driven perspective. On the other hand, mechanistic models may provide useful guidelines for the development of more efficient, consistent, and robust learning algorithms in epidemic applications and can be used as a validating framework for machine learning [203]. A new modeling paradigm for cholera may be established by integrating classical epidemic models and machine learning techniques, which, if successful, can potentially lead to new breakthrough in the study of cholera dynamics.

Another useful approach is based on ensemble modeling and forecasting [204], where multiple models, often constructed by independent research groups, are implemented and their predictions are combined to guide decision making. The ensemble framework allows different types of models (ODEs, PDEs, stochastic systems, agent-based models, machine learning techniques, and others), based on different assumptions, to be developed for answering the same questions, with a goal of better capturing the complete range of possible outcomes than a single model does. The ensemble procedure can be repeated at regular intervals in the course of an epidemic. Ensemble modeling has been conducted for the COVID-19 pandemic with robust projections [205,206]. This modeling approach would also be applicable to cholera outbreaks, and open communication and collaboration between different cholera-modeling groups would be crucial for its successful application.

Future modeling efforts for cholera may also benefit from a holistic modeling framework that connects relevant biological, ecological, epidemiological, immunological, and societal processes, provided that sufficient and high-quality data are available from these different fields. Models generated from such a framework will most likely be multi-scale, strongly nonlinear, and highly complex. Consequently, advanced numerical methods and data analysis techniques will be needed, facilitating a comprehensive and computationally intensive modeling approach. To achieve this goal, interdisciplinary collaboration should be strongly encouraged and promoted, and such collaborative work may involve epidemiologists, mathematical modelers, computational scientists, ecologists, microbiologists, immunologists, and social scientists.

## Data Availability

Not applicable.
